# Effects of passive smoke inhalation on the vocal cords of rats

**DOI:** 10.1016/S1808-8694(15)30057-4

**Published:** 2015-10-19

**Authors:** Josilene Luciene Duarte, Flavio Augusto Cardoso de Faria, Danielle Santi Ceolin, Tania Mary Cestari, Gerson Francisco de Assis

**Affiliations:** a Hearing and Speech Therapist specialized in Audiology; b PhD Professor of Physiology at the Department of Biologic Sciences of the Dentistry School of Bauru - USP).; c Biologist and Laboratory Technician for the Histology program of the Department of Biologic Sciences of the Dentistry School of Bauru - USP).; d Biologist enrolled in the PhD program on Buccal Biology at the Dentistry School of Bauru - USP and Laboratory Technician for the Histology program at the Department of Biologic Sciences of the Dentistry School of Bauru - USP).; e Professor of Histology of the Department of Biologic Sciences of the Dentistry School of Bauru - USP.

**Keywords:** vocal fold, rats, smoke, tobacco

## Abstract

Few studies have demonstrated the pathologic reactions yielded by smoke inhalation on the airway in rats. **Aim:** The aim of this study was to analyze the possible histopathological effects produced by chronic cigarette smoke inhalation on the vocal folds of rats. **Study design:** Experimental. **Material and Method:** 36 male rats (Rattus norvergicus Wistar strain), aged 60 days, were kept in cages and exposed to inhalation of the smoke produced by 10 cigarettes lit 3 times a day, 7 days a week, for periods of 25, 50 and 75 days, and their respective controls. Thereafter the animals were killed and their larynxes were dissected and submitted to histological processing for achievement of histological sections, which were stained with Hematoxylin and Eosin and analyzed by light microscopy. **Results:** The rats exposed to smoke displayed smaller (p< 0,05) body mass than the control group. There was hyperplasia and squamous metaplasia in the free edge of the vocal fold and squamous hyperplasia on the middle portion of the vocal fold in all 3 study periods. Moreover, the 50-day group revealed keratinizing metaplasia in this area. Morphological alterations in other areas of the larynx and inflammatory reaction of the lamina propria were also not observed. **Conclusion:** It was concluded that the passive inhalation of cigarette smoke yields important morphological changes in the vocal fold epithelium, which may progress to neoplasia.

## INTRODUCTION

Despite medical advances in understanding the mechanisms of aggression caused by tobacco smoking in humans, cigarette smoking is still the main cause of early death in the United States, and is the major factor for occurrence of various diseases (Sherman, 1991)^1^. In the year 2030, smoking may be the main isolated cause of deaths and it could cause 10 million deaths yearly (Menezes et al. 2002)^2^. Thus, anti-smoking campaigns are the priority for international organizations such as the World Health Organization (WHO, 1997)^3^. In this beginning of century, not only scientific papers, but also the press has been frequently approaching focusing this topic.

According to the International Union against Cancer (IUAC), approximately 95% of larynx cancer cases are related to smoking, and the probability increases when associated to alcohol (Franceschi et al. 2000; Levi, 1999)^4^, ^5^. This type of cancer is easily diagnosed and highly curable when in early stages, where persistent hoarseness is the first symptom.

More than 400 chemical substances are isolated from cigarette smoke, among which 250 are generated from processed tobacco, while the remaining ones represent the addition of pesticides, organic and metallic compounds (Dube e Green, 1982)^6^. Relatively stable free radicals are found in the particle phase which can reduce the oxygen molecule to peroxide and, occasionally, may produce hydrogen peroxide and hydroxylic radicals (Churg e Pryor, 1985; Nakayoma et al., 1984)^7^, ^8^. Radioactive components are also present both in tobacco and cigarette smoke, including lead (210 Pb) and polonium (210 Po), which contribute to the tobacco carcinogenic potential (Block e Bretthaaver, 1968; Cruze, 1984)^9^, ^10^. Various other toxic substances, such as carbon monoxide, may also be found.

Among the damages caused by smoking, its carcinogenic potential is surely one of the most important aspects. Cigarette smoke contains agents which may contribute to the carcinogenesis transformation, initiating, promoting or accelerating its harmful effects (DHHS-US, 1982)^11^.

The histopathological manifestations which most authors mention for the smokers’ airways are secretory hyperplasia and hypertrophy. These alterations may be interpreted as an adaptive response of the airway when submitted to chronic aggression. Thus, the presence of a thicker mucus layer would provide protection against the passage of irritating agents to the respiratory epithelium (Cendon, 1997)^12^.

Cigarette smoke is considered a larynx tumor inducer in hamsters (Homburger, 1975)^13^ and at least one study has shown a 1% to 9% increase in pulmonary adenomatose tumor cases (Dalbey et al. 1980)^14^. On the other hand, this type of tumor is different from the squamous carcinoma previously observed in human smokers.

Little has been done to observe the pathological reactions induced by inhalation of cigarette smoke in rats and their whole airways, including the larynx. In a study carried out by Haussmann et al. (1998)^15^, the larynx was considered the organ most sensitive to histopathological changes after exposure to cigarette smoke. There is dose-dependent diffuse squamous metaplasia of the pseudostratified epithelium and squamous hyperplasia on the base of the epiglottis.

Even though some studies show morphologic data on animal larynxes as an experimental model of smoke exposure, more studies are necessary to better characterize these alterations.

This study aims at making a subjective morphologic analysis of the possible histopathological effects on the larynx of rats subject to chronic inhalation of cigarette smoke, especially on the vocal folds, through light microscopy.

## MATERIALS AND METHODS

Thirty three male rats (Rattus norvergicus Wistar) aged 60 days and with body mass around 180 grams from the FOB/USP animal lab were included in this study. Groups of six animals were kept in cages cleaned up every two days under similar light and temperature conditions, and receiving water and food “ad libitum.”

Three groups of six animals were exposed daily to the inhalation of smoke produced by ten cigarettes lit in the morning, afternoon and evening, in a total of thirty cigarettes/day, for a whole week (Cendon et al. 1997)^12^. The animals in each group were slaughtered 25, 50 and 75 days after the exposure, respectively. The control groups were slaughtered at the same time as each study group.

The inhalation system used consists of a wooden box measuring 28x38x48cm which was divided in two compartments by a metal screen. One compartment was used for the rats, and the other for the cigarettes. A respiratory pump, model 16/24, C.F.Palmer (London), was fitted to the wooden box and emitted an air flux of 450ml/min, while the cigarettes were kept lit for at least 10 minutes. Small holes for air drainage were made on the animal compartment, allowing the smoke to escape.

The cigarette brand used was Derby, Kingsize, due to its leading ranking in cigarette sales, probably because of its low cost and high levels of tar. (tar - 12mg; nicotine - 0.9mg; carbon monoxide - 12mg).

After exposure, the animals were given anesthesia and their body masses were measured on a precision scale. Their larynxes were removed and submerged in phosphate buffered formalin 10% for 24 h at room temperature. Anesthesia was reached with 0.1 ml of Dopalen (Ketamine chloridrate) + 0.1ml of Anasedan (xylazine chloridrate) + 0.1ml of atropine sulphate. After fixation, the larynxes were submitted to histological processing with dehydration in ethanol, dyafanization in xylol and embedded in resin for cross sectioning. Before this procedure, the specimens were kept in a demineralizing agent (EDTA) for 10 days, since the pilot study had shown mineralization on the ventral portion of the thyroid cartilage. After that, 5µm thick semi consecutive sections were obtained and stained with hematoxylin and eosin (H.E.) and analyzed through light microscopy, comparing the study groups and the controls, the differential morphological aspects, mainly the connective tissue of the epithelium (lamina propria) of the larynx mucosa, especially of the vocal folds.

## RESULTS

### Body Mass

The statistical analysis of the results on table 1 shows that the animals in the study groups had body mass 14%, 15% e 19% (p <0.05) below the respective controls of groups after 25, 50 and 75 days following exposure.

**Table 1:** Body mass (g) of the rats, according to the experimental study period (days) and respective controls.

Periods	Control (a)	Experimental (b)	Axb
25	301.82 ± 18.20*	261.23 ±28.18*	P < 0.03**
50	322.46 ± 36.07*	276.02 ± 43.97*	P < 0.01**
75	350.45 ± 23.94*	284.05 ± 15.79*	P < 0.01**

Mean and standard deviation (n=6)

Level of statistical probability (p < 0,05)

### Morphological description

 

#### Control

**Figure 1:**
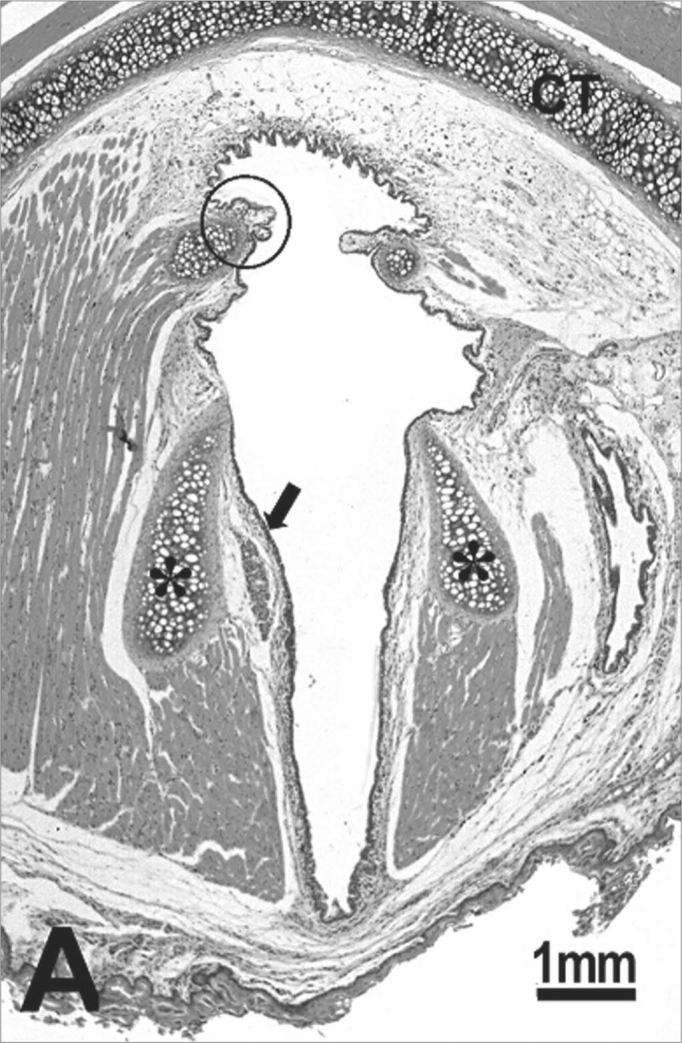

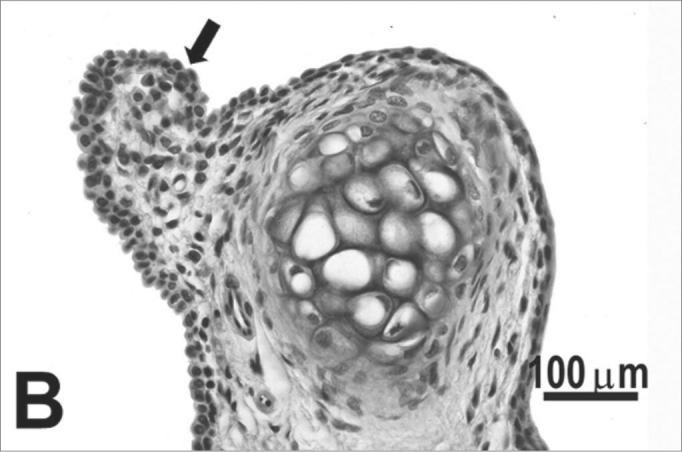
Control group: **A:** overview of the larynx demonstrating the thyroid (CT) and arytenoid (asterisk) cartilages, muscles, connective tissue, seromucous gland, free edge of the vocal fold (circle) and middle portion of the vocal fold (arrow); **B:** cuboidal epithelium (arrow) of the free edge of the vocal fold; C: stratified squamous epithelium (arrow) of the middle portion of the vocal fold. H.E.

The histological sections of the animals in the control group show that the glottis region of the rat is formed by three types of epithelium. The ventral portion epithelium is pseudostratified ciliated and the lamina propria shows a loose connective tissue composed of few collagen and elastic fibers, while fibroblasts, mast cells and macrophages are the most evident cells. A small stratified columnar epithelium is observed close to the free extremity of the vocal folds (figure 1B), and the lamina propria reveals a more dense connective tissue followed by perichondrium. The middle portion of the vocal folds (Figure 1C), close to the arytenoid cartilage, is composed of stratified squamous epithelium, which is thinner on the superior portion of the cartilage and thicker on the middle portion; the lamina propria around this area is formed by loose connective tissue composed of few collagen fibers, elastic fibers, fibroblasts, blood vessels and some macrophages. The dorsal portion of the glottis shows a stratified epithelium which might be cuboidal or squamous depending on the area analyzed, and the connective tissue in the lamina propria is denser. On the frontal portion this area showed thyroid lamina and arytenoid cartilage, skeletal muscle fibers, nerve fiber bundles and seromucous glands (Figure 1A).

#### Study group - 25 days

**Figure 2:**
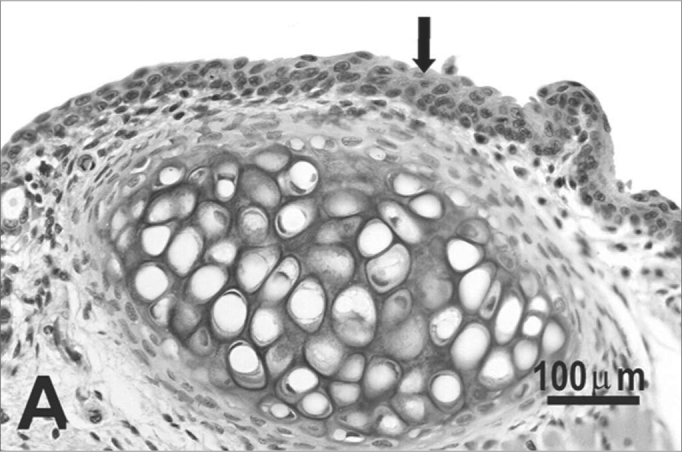

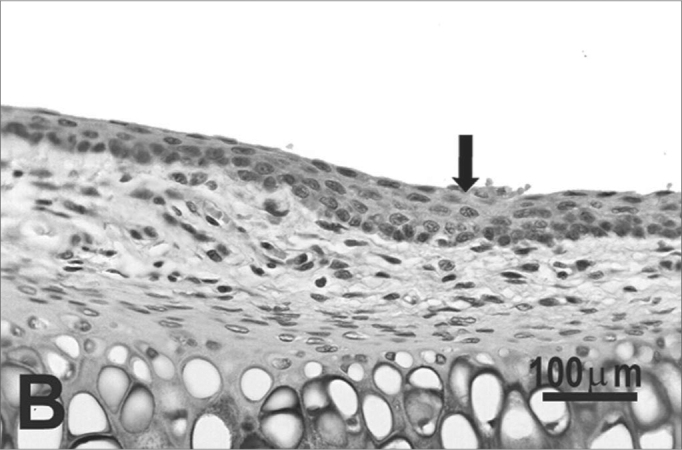

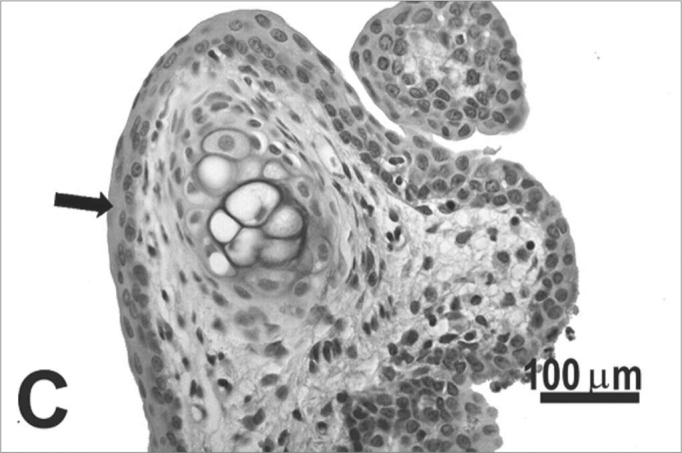

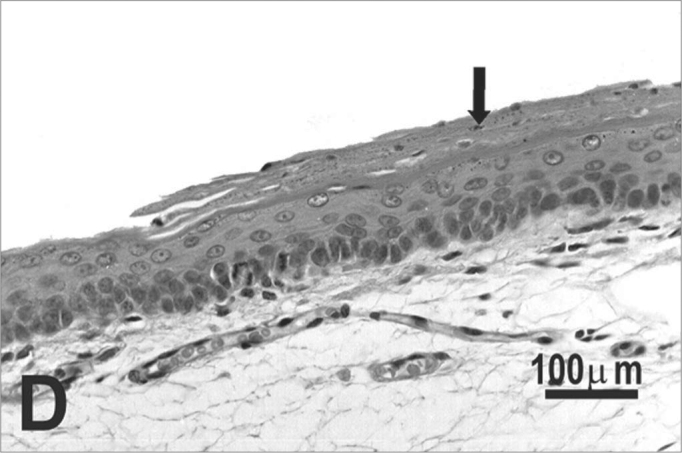

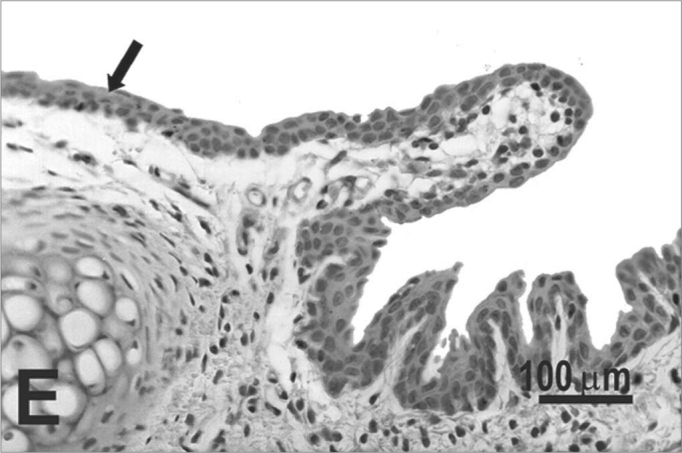

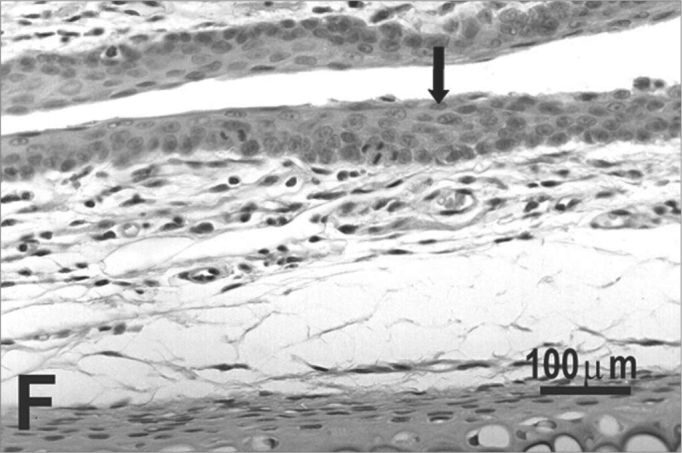
Vocal folds of rats submitted to passive smoke inhalation. **A:** squamous metaplasia (arrow) of the free edge of the 25-day group; **B:** mild hyperplasia (arrow) of the middle portion of the 25-day group; **C:** metaplasia and hyperplasia (arrow) of the free edge of the 50-day group; **D:** keratinizing metaplasia and hyperplasia (arrow) of the middle portion of the 50-day group; **E:** metaplasia and hyperplasia of the free edge (arrow) of the 75-day group; **F:** hyperplasia of the middle portion of the 75-day group. H.E.

The type of epithelium and the lamina propria were similar to the 25 days control group in almost all areas examined in the larynx. On the other hand, the animals exposed to cigarette smoke showed metaplasia associated to moderate hyperplasia on the free border of the vocal folds, i.e. the epithelium changed from cuboidal to squamous (figure 2A) and showed a moderate hypertrophy in the middle portion of the vocal folds, close to the arytenoid cartilage (figure 2B). No congested vessel or inflammatory cell increase was observed on the connective tissue of these animals.

#### Study group - 50 days

No changes were observed on the epithelium or lamina propria in both ventral and dorsal regions on the larynxes of the animals in this group. The free border of the vocal folds showed the same type of squamous metaplasia observed on the animals from the 25 days study group (figure 2C). The most noticeable change, though, was the presence of squamous hyperplasia on the middle portion of the vocal folds (also observed on the 25 days study group), and metaplasia with keratinization on this area of the epithelium (figure 2D).

Congested blood vessels were observed throughout the larynx lamina propria, but without the presence of an inflammatory infiltrate.

#### Study group - 75 days

Similarly to the animals of groups 25 and 50 days, no change was observed over the type of epithelium or changes on the lamina propria of the ventral or dorsal areas. The hyperplasia of the free border of the vocal fold was similar to the one observed on the study groups of 25 and 50 days. Other results include moderate hyperplasia and metaplasia of the epithelium on the superior portion of the arytenoid cartilage (figure 2E) and middle portion of the vocal folds (figure 2F). Metaplasia with keratinization and congested blood vessels were observed on the 50 days study group, but these couldn’t be found on the 75 days study group.

## DISCUSSION

Cigarette is, nowadays, one of the most damaging agents to humans leading to both physical and psychological addictions. Ending the habit of smoking requires treatments for the elimination of both the physical and psychological dependences on nicotine. Besides that, some of the 400 substances found in cigarettes are suspected of causing genetic changes. One of such substances is tar, considered a highly carcinogenic agent and responsible for the highest incidence of lung, mouth, larynx, esophagus, stomach, prostate, bladder and colon cancers (Dube e Green, 1982,; Coelho, 2001)^6^, ^16^.

Around 30% of the deaths by cancer registered are due to smoking, being distributed in: 90% of the deaths from lung cancer; 97% of deaths from larynx cancer; 25% of deaths from heart disease; 85% of deaths from bronchitis and emphysema; 25% of deaths from stroke and 50% of death cases due to skin cancer. This happens because of early ageing of cells in the organism because of reduced oxygen supply through the blood (5% less) and the following increase in free radicals (Coelho, 2001)^16^.

The present study has exposed the animals to smoke from ten cigarettes (three times a day) and has shown its effects, especially on the larynx. The rat larynx has been highly used as an experimental model for speech, since its shape is similar to the human larynx, allowing some comparisons of its vocal behaviors. According to Kurita et al. (1983)^17^, there are differences on the membranous portion and the densities of the vocal folds of humans and rats, but the lamina propria of both is divided in three portions, that is, a superficial layer with few fibrous components, an intermediary layer with more components and a deep layer with more collagen and elastic fibers. The division between the surface and the intermediary portion is not obvious, but the transition between the intermediary and the deep layers is clear.

Some investigations (Lee et al. 1992; Haussmann et al. 1998)^18^, ^15^ report that smokers gain less body mass than non-smokers. On the other hand, studies that effectively demonstrate this difference between non-smokers and passive smokers are difficult to find. In the present study, the animals exposed to cigarette smoke show a body mass slightly lower than the control group, and this difference has increased with the study periods ranging from 14% to 19%. Lee et al. (1992) evaluated rats exposed to cigarette smoke for six hours a day over a period of 14 days in conditions similar to the ones in our study and hasn’t observed significant differences in body mass. In 1998, Haussmann et al. prepared a study similar to the one by Lee et al. (1992) and observed a statistically significant difference in body mass after 59 days of exposure. The authors submit that after the final period, all the rats gained body mass at the same rate, indicating reversion of the effects caused by passive inhalation of cigarette smoke.

Another aspect highly discussed among investigators who study the larynx is the distribution of its epithelium, since there are various types of epithelium according to the localization of the glottis. Three different types of epithelium were observed in cross sections of the larynx where the vocal fold is located, being stratified squamous on the middle portion of the vocal fold and dorsal aspect of the larynx, pseudostratified columnar epithelium on the dorsum and the free extremity of the vocal fold, and columnar pseudostratified ciliate epithelium on the ventral portion of the larynx. In 1977, Smith^19^ investigated the vocal folds of rats and noticed the presence of stratified epithelium and non ciliated columnar pseudostratified epithelium, the latter being more frequently observed and also found on the dorsal aspect of the vocal fold. Walker et al. (1978)^20^ observed different transitions between various types of epithelium. The non-ciliated columnar pseudostratified epithelium was observed on the area close to the thyroid cartilage, including the vocal fold free border, changing to stratified squamous epithelium on the middle portion which is thinner in this region and thicker in the dorsal region. In a recent study, Marcelino et al. (2000)^21^ found two types of epithelium in the larynx, stratified squamous in the area close to the thyroid cartilage and vocal folds and ciliated pseudostratified epithelium in the other regions.

A consensus is needed to classify the type of epithelium found in each area of the larynx, since the regions are very small and allow multiple transitions among them. The different classifications described by the authors might be due to variations in thickness of the histological sections or even due to the position of the larynx when embedded, since the vocal folds of rats are organized diagonally in the larynx and these should be embedded in a similar position.

The histopathological changes observed were the hyperplasia and metaplasia of the vocal fold. We suppose the initial point of metaplasia alteration is the columnar epithelium observed in the free edge of the vocal fold, since it could be observed 25 days after exposure to smoke in all rats examined and also the other periods; metaplasia was only found on the middle portion after 50 days. The hyperplasia was observed on the three study periods and in a crescent fashion.

Squamous metaplasia was observed in the free edge and keratinizing metaplasia was found in the middle portion. The squamous metaplasia was associated to a susceptibility to the development of “in situ” carcinoma and invasive squamous carcinoma (Trump et al. 1984; Spencer e Couldery, 1985)^22^, ^23^. It should be emphasized that hyperplasia increased during the three stages of the study, as well as the squamous metaplasia; the keratinizing metaplasia was observed only in the 50 day group, and couldn’t be found in the 75 day group. This could be explained by the supposition that the epithelium might have stabilized to reach protection.

The type of alteration observed was similar to the ones seen in previous studies. In 1979, Meade et al.^24^ found squamous metaplasia in the larynx of rats six weeks after exposure to cigarette smoke. The authors report, though, that this could happen earlier in vitamin A deficiency. Haussmann et al. (1998)^15^, in a study similar to the present one, but using three different concentration of carbon monoxide (6, 13 e 28 ppm), observed that hyperplasia and squamous metaplasia in the free edge of the vocal fold was not dosage dependent, considering, thus, that the hyperplasia of the epithelium in the middle portion was.

The transition from squamous epithelium to respiratory epithelium in the larynx is the most sensitive to development of cellular alterations such as degeneration, squamous metaplasia or hyperplasia, when attacked by inhalation of xenobiotics (Gopinath et al. 1987; Lewis, 1981)^25^, ^26^. The areas of hyperplasia and metaplasia found in the present study were more visible in the middle portion and the free border of the vocal fold, followed by the trachea, and the morphological analysis revealed that the area immediately underneath them showed respiratory epithelium.

The human larynx is constituted of pseudostratified columnar ciliated epithelium, with squamous stratified epithelium in the vocal fold. As the primary goal of this study was to check the potential histopathological modification affecting the vocal folds of rats after exposure to cigarette smoke, this investigation can be considered a warning to society, once the epithelium of both is of the same kind, thus the pathological alterations might also be the same.

## CONCLUSION

According to the results found, we can conclude that the passive inhalation of cigarette smoke leads to morphological alterations in the larynx of animals, especially the vocal folds, leading to increased risk of neoplasia.
